# Maternal Weight Management to Prevent the Developmental Programming of MAFLD in Offspring of Obese Mothers

**DOI:** 10.3390/nu15092155

**Published:** 2023-04-30

**Authors:** Amanda Renae Purcell, Sarah Jean Glastras

**Affiliations:** 1 Kolling Institute of Medical Research, Sydney 2065, Australia; amanda.purcell@sydney.edu.au; 2 Faculty of Medicine and Health, The University of Sydney, Sydney 2006, Australia; 3 Department of Diabetes, Endocrinology and Metabolism, Royal North Shore Hospital, Sydney 2065, Australia

**Keywords:** maternal obesity, NAFLD, fetal programming, weight loss, GLP-1 receptor agonist, developmental origins of health and disease (DoHAD), hepatic steatosis

## Abstract

The global surge of obesity amongst women of reproductive age has raised concerns surrounding the health consequences for their offspring as there is a formidable link between an obesogenic maternal environment and the developmental programming of metabolic dysfunction in the offspring. Specifically, the offspring of mothers with obesity have a three-fold higher risk of developing metabolic-associated fatty liver disease (MAFLD) compared to the offspring of healthy-weight mothers. Given the burgeoning burden of obesity and its comorbidities, it is essential to focus research efforts on methods to alleviate the intergenerational onset of obesity and MAFLD. This review summarizes the current research surrounding the developmental programming of MAFLD in the offspring of mothers with obesity and examines the potential for weight interventions to prevent such metabolic dysfunction in the offspring. It focuses on the benefits of pre-pregnancy interventional strategies, including dietary and exercise intervention, to ameliorate adverse liver health outcomes in the offspring. The utility and translation of these interventions for humans may be difficult for prospective mothers with obesity, thus the use of pre-pregnancy therapeutic weight loss aids, such as glucagon-like peptide-1 receptor agonists, is also discussed.

## 1. Introduction

In recent decades, global obesity rates have grown to pandemic proportions. According to the World Health Organization (WHO), in 2016 over 650 million adults had obesity, defined as a body mass index (BMI) >30 kg/m^2^ [1]. The rate of maternal obesity is rising in parallel, with data from the US National Health and Nutrition Examination Survey (NHANES) revealing that among American women aged 20–39 years of age, more than 30% have obesity [2]. Maternal obesity influences both maternal and offspring health; it is associated with an increased risk of adverse pregnancy-related conditions including gestational diabetes, pre-eclampsia, and stillbirth, and the offspring born to mothers with obesity have higher rates of obesity from childhood to adulthood [1,3,4]. The burgeoning impact of obesity poses a significant risk for increased morbidity and mortality, particularly in relation to the metabolic complications of obesity, including type 2 diabetes (T2D) and prediabetes, chronic kidney disease (CKD), cardiovascular disease (CVD), and metabolic-associated fatty liver disease (MAFLD) [5].

MAFLD, previously referred to as non-alcoholic fatty liver disease, is a term encompassing a range of diseases caused by the accumulation of lipid products in the liver [6]. MAFLD affects more than a quarter of the global population, with its increase in prevalence being linked to rising rates of obesity [7]. MAFLD is the leading cause of end-stage liver disease, a disastrous consequence that can lead to premature death unless liver transplantation can be achieved [8]. Histologically, MAFLD is characterized by steatosis, which is defined as intrahepatic triglyceride content >5% of the total liver volume [9]. Beyond steatosis, metabolic-associated steatohepatitis (MASH) can develop, manifesting as simple steatosis concurrent with inflammation, oxidative stress, and fibrosis, ultimately leading to cirrhosis, and an increased risk of liver failure and malignancy [10]. Since the early stages of MAFLD commonly show no apparent symptoms, the identification of individuals with a predisposition for MAFLD may enable the intervention and prevention of metabolic liver disease. Maternal obesity has been well established as a risk factor for metabolic dysfunction and obesity in offspring. More recently, its impact on the progression of MAFLD has been elucidated, with several pre-clinical and clinical models linking a maternal obesogenic environment with higher rates of MAFLD in adult offspring [11,12,13,14,15]. Considering the escalating rate of obesity amongst reproductive-age women, it is crucial to devise interventional strategies in the perinatal period to mitigate the established risk of MAFLD for the unborn offspring of mothers with obesity.

## 2. Molecular Mechanisms of Obesity and Its Influences on Normal Liver Function

Overnutrition, which is characteristic of obesity, promotes lipid storage in adipocytes in the body. When adipose tissue can no longer accommodate the increase in lipid products, it results in adipocyte hypertrophy, leading to a plethora of physiological alterations including systemic fatty acid (FA) flux, adipokine secretion and adipocyte apoptosis [16]. Adipocytes are highly responsive to insulin. In normal physiological conditions, insulin promotes the formation of triglycerides (TGs) from FAs by lipogenesis and inhibits their breakdown by lipolysis. However, in the case of obesity-induced insulin resistance, these cells no longer respond to insulin’s metabolic effects and the systemic FA flux is exacerbated as insulin can no longer inhibit lipolysis. These adaptations to adipose tissue physiology negatively impact the liver as it increases the availability of lipid products in the blood, leading to ectopic fat uptake in the liver. As such, individuals with obesity and MAFLD have high levels of insulin resistance [17]. 

**Figure 1:**
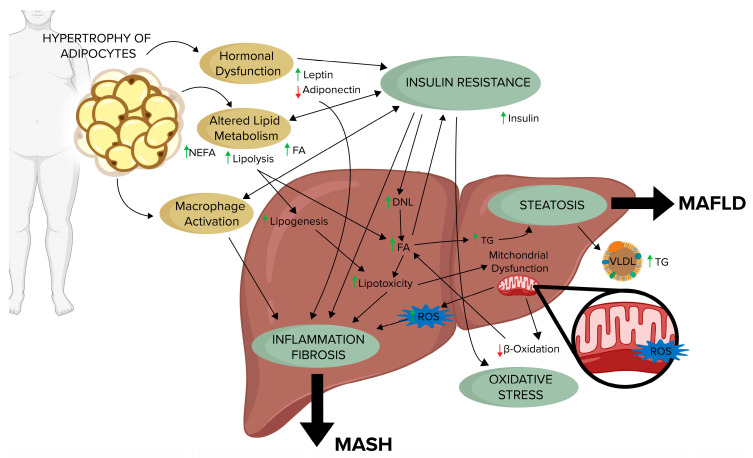
Obesity induces adipocyte dysfunction leading to an increased flux of systemic lipid products which is worsened by insulin resistance. Further, adipose tissue dysfunction causes hormonal dysregulation of leptin, adiponectin, and the activation of inflammatory pathways. The increase in systemic lipid availability causes the ectopic uptake of fats by the liver, and insulin resistance stimulates de novo lipogenesis, together causing steatosis, characteristic of MAFLD. Lipotoxicity in the liver causes mitochondrial dysfunction and oxidative stress which progressively leads to the onset of fibrosis in the liver, characteristic of MASH. NEFA, non-esterified fatty acids; FA, fatty acids; DNL, de novo lipogenesis; TG, triglycerides; VLDL; very low-density lipoproteins; MAFLD, metabolic-associated fatty liver disease; MASH, metabolic-associated steatohepatitis; ROS, reactive oxygen species. An increase is indicated by green arrows and a decrease is indicated by red arrows.

The liver assumes a central metabolic role, acting as an intermediary organ between exogenous and endogenous energy sources. The liver uptakes systemic lipid products in the form of FAs as well as post-prandial non-esterified fatty acids (NEFAs). FAs are also produced in the liver by de novo lipogenesis [18]. These lipid species are then esterified to form TGs and stored transiently in hepatocytes either for downstream energy expenditure via mitochondrial β-oxidation or systemic secretion within very low-density lipoproteins (VLDLs). In the over-satiated state of obesity, the increase in the availability and production of lipid species causes the rate of FA acquisition in the liver to be disproportionately higher than the rate of FA output, driving the accumulation of lipotoxic lipid species which is a key component of steatosis [19]. These adaptations can cause the phenotypic changes of MAFLD (Figure 1).

Hormonal dysregulation is a hallmark feature of obesity. Alongside insulin resistance, alterations in the activity of the adipose tissue-derived hormones, leptin and adiponectin, contribute to metabolic dysfunction in the liver. Leptin mediates FA metabolism and conversely, adiponectin decreases lipogenesis and glucose output in the liver in addition to regulating inflammation [20]. High levels of leptin and low levels of adiponectin are associated with an increased risk of liver damage in individuals with MAFLD.

When obesity goes unmanaged, an intrahepatic inflammatory process begins and the progression from MAFLD to its more serious stage of MASH occurs. Inflammation is an essential immune response that allows the reparation of injured tissues and protection against foreign pathogens [21]. However, the persistent inflammatory response that accompanies obesity can be detrimental in the context of MAFLD, as it can aggravate liver injury. The hypertrophy of adipocytes results in the activation of pro-inflammatory macrophages that secrete cytokines and activate inflammatory pathways making it the predominant source of systemic inflammation [22]. These cytokines also have receptors in liver parenchyma that induce signaling cascades involved in insulin resistance. Inflammation directly influences insulin resistance, and, reciprocally, insulin resistance exacerbates inflammation [23]. The activation of systemic macrophages together with the activation of resident liver macrophages, called Kupffer cells, results in the systemic and intrahepatic accumulation of inflammatory mediators which has deleterious effects on liver function and drives the progression of MASH [24]. 

Alongside inflammation, mitochondrial dysfunction and resultant oxidative stress contribute to this disease. Oxidative stress arises from a discrepancy between the balance of reactive oxygen species (ROS) generation and antioxidant defenses. Oxidative stress can trigger damage to hepatocytes through the alteration of lipids, proteins, and DNA contents. Oxidative stress, together with inflammation can lead to the progression of liver fibrosis [25]. Fibrosis is the reparative response to chronic liver damage where hepatic stellate cells (HSCs) activate and deposit extracellular matrix proteins, such as collagens, at the site of injury [26]. In a normal setting, fibrosis preserves tissue stability during injury; however, the sustained and progressive fibrosis that is seen in MASH has pathogenic features. Fibrosis distorts the liver’s architecture over time which may lead to cirrhosis and malignancy.

## 3. Developmental Programming of Metabolic Dysfunction in the Offspring of Mothers with Obesity

The hallmark features of obesity-induced metabolic dysfunction, namely hormonal and lipid dysregulation, are relevant to both mothers with obesity and their offspring, leading to the intergenerational progression of MAFLD and associated liver diseases. Although genetics and postnatal environment play an eminent role in the prediction of long-term health, an obesogenic maternal environment also fosters adverse fetal effects that influence health from birth through to adulthood [27]. This concept is known as the developmental origins of health and disease (DOHaD), suggesting that an adverse intrauterine environment is crucial in shaping an individual’s long-term health [28]. Epidemiological studies have shown that offspring born to mothers with obesity are at risk of increased birth weights, heightened adipose tissue mass, and childhood obesity [29,30]. This risk spans into adulthood, as childhood body weight is positively correlated with adulthood body weight. A high maternal pre-pregnancy BMI is also associated with known constituents of metabolic dysfunction in the offspring, namely increased insulin resistance which can exacerbate adipose tissue lipolysis and increase the hepatic uptake of lipid species [13,14,29,31]. 

### Evidence for an Association between Maternal Obesity and an Increased Risk of MAFLD in Offspring

Although discerning the impact of developmental programming and post-natal environment is difficult in human studies, several longitudinal cohort studies have linked maternal pre-pregnancy BMI with MAFLD risk in the offspring. The Raine cohort study found an association between a maternal pre-pregnancy BMI >30 kg/m^2^ with an increased risk of MAFLD in the offspring at 14 years of age [32]. Similarly, data from the ESPRESSO cohort study found a three-fold higher risk of MAFLD in the offspring of mothers with obesity compared to the offspring of mothers with a healthy body weight [11]. Other cohort studies found that maternal pre-pregnancy obesity was strongly associated with increased hepatic steatosis in adolescent offspring, independent of childhood or adolescent adiposity [33,34].

There is also overwhelming evidence of the developmental programming of MAFLD in offspring in preclinical animal studies. In a model of maternal obesity in Japanese Macaques, fetal hepatic steatosis was higher in late gestation in the offspring of mothers fed a high-fat diet (HFD) compared to the offspring of lean mothers, implicating maternal overnutrition in fetal hepatic fat accumulation [13]. Further, the elevation of expression of genes involved in lipid homeostasis, namely sterol regulatory element-binding protein-1c (SREBP-1c), was observed in offspring of HFD-fed mothers, and this was further increased when offspring were exposed to postnatal HFD [12]. Insulin activates hepatic SREBP-1c to regulate de novo lipogenesis in the liver and its heightened expression is indicative of steatosis [35]. In this same model, the offspring also had primed expression of genes involved in oxidative stress and inflammation. Furthermore, a model of maternal obesity in C57Bl/6J mice demonstrated that exposure to perinatal HFD increased the expression of profibrotic genes in the liver of offspring that were fed a postnatal low-fat diet [36]. It was found that fibrosis was accelerated in the offspring of mothers with obesity when the offspring were reared on a postnatal HFD [37]. 

The developmental programming of MAFLD in the offspring of mothers with obesity is thought to be attributed to changes in epigenetic profiles in the placenta and the liver of the offspring. The epigenome refers to DNA and histone modifications that alter genetic transcription and cellular function [38]. MicroRNAs (miRNAs) are epigenetic regulators that have been shown to alter the expression of genes involved in lipid metabolism, oxidative stress, and inflammation in the liver of humans [39,40]. Furthermore, a study assessing the impact of an obesogenic western-style diet (WSD) in baboons found that at late gestation, the mRNA expression of miR-182-5p and miR-183-5p was downregulated in the placenta of mothers fed WSD, concomitant with alterations in pathways involved in lipid metabolism and inflammation [41]. In a mouse model of maternal obesity, the newborn offspring of mothers with obesity had upregulated levels of the miRNA *Let-7* in the liver, which regulates hepatic AMP-activated protein kinase a2 (AMPKa2) protein expression [42]. A knockout mouse model of AMPKα2 was shown to have significantly increased hepatic steatosis, inflammation, and fibrosis [43]. Hence, the upregulation of *Let-7* may be implicated in the progression of MAFLD through the AMPKa2 pathway [44]. Furthermore, a mouse model of maternal obesity concluded that the programming of liver perturbations in the offspring may be catalyzed by DNA hypermethylation, an epigenetic alteration that results in the downregulation of gene expression. In this study, maternal obesity resulted in the hypermethylation and downregulation of the zinc finger transcription factor, Miz1, in the fetal liver [45], which has been shown to reduce hepatocyte inflammation [46]. Given the clear evidence of the adverse liver health outcomes catalyzed by the epigenetic modifications induced by maternal obesity, the optimization of maternal health in the perinatal period may be beneficial to mitigate this risk for the offspring.

## 4. Maternal Weight Management to Mitigate Adverse Health Outcomes in Offspring

Clinical studies focusing on maternal weight management during pregnancy have yielded poor results regarding offspring health outcomes. Both the LIMIT and UPBEAT trials investigated the benefits of lifestyle modification during pregnancy, finding no effect of gestational weight management programs on neonatal outcomes [47,48]. There are known benefits of pre-conception weight management for maternal health, fertility, and pregnancy outcomes [49]; however, whether or not these benefits extend to the offspring is largely unexplored.

In contrast to human studies, some preclinical investigations have found a benefit of pre-pregnancy weight management in models of maternal obesity that demonstrate beneficial effects on offspring health. A rodent study investigating maternal pre-pregnancy exercise improved insulin resistance in male offspring [50]. Similarly, a study examining pre-pregnancy and during-pregnancy exercise intervention found that offspring born to exercised mothers had less susceptibility to insulin-resistant diseases compared to those born to sedentary mothers [51]. 

More specifically, preclinical models have proven the efficacy of maternal pre-pregnancy weight loss via lifestyle changes in improving metabolic liver health in their offspring (Table 1). Briefly, in a model of maternal obesity in Japanese Macaques, dietary intervention 9 weeks prior to conception reduced lipogenic gene expression and oxidative stress in the fetal liver in the third trimester. Interestingly, a study comparing the timing of dietary intervention in a mouse model of maternal obesity found that dietary intervention only 1 week prior to conception did not alleviate the risks associated with maternal obesity for the offspring as they exhibited disrupted insulin sensitivity in hepatocytes, increased hepatic expression of SREBP-1c and hepatic steatosis, like the offspring of mothers with obesity. However, similar to the study in Japanese macaques, this was avoided in the offspring of mothers with dietary intervention 9 weeks prior to conception, suggesting that early maternal dietary intervention is effective in reducing the risk of MAFLD in the offspring of mothers with obesity [52]. Animal studies investigating maternal exercise in the prenatal period have yielded similar results; maternal exercise prior to conception in rodent mothers with obesity was shown to reduce hepatic fibrosis and regulate the expression of genes involved in β-oxidation and lipogenesis in the liver of the offspring [53,54].

Ultimately by reducing the transgenerational risk of obesity via maternal weight management in the prenatal period, the burden of obesity-related metabolic conditions could be reduced in the offspring. From the pre-clinical evidence of improved metabolic liver health in the offspring, it is plausible that pre-conception lifestyle interventions are necessary to reduce the risk of MAFLD and its precursors in the offspring of mothers with obesity, highlighting that the method and timing of the intervention have a great impact on health outcomes.

### 4.1. Challenges for Maternal Weight Modification Via Lifestyle Interventions 

Women with obesity are advised to attempt a reduction of 5–7% of body weight prior to conception [56]. Importantly, lifestyle modification typically requires 6–12 months to reduce body weight by 5–10%, however, survey data revealed that more than 80% of overweight and obese women are unwilling to postpone pregnancy plans longer than 3 months to achieve such weight reductions [57]. Furthermore, sustaining weight loss beyond initial loss is challenging, and weight regain is commonly experienced by many people undertaking weight loss intervention [58]. It was found that pre-pregnancy lifestyle intervention in women with obesity produced modest weight reductions incongruous with the recommended weight loss levels from key international guidelines [59]. As such, they led to minimal benefit on perinatal outcomes. In contrast, nutritional deficiencies from severe dietary restriction to achieve pre-pregnancy weight loss can have lasting negative impacts on offspring health. For example, offspring born to mothers during the Dutch famine, and the offspring of mothers who had bariatric surgery within 1 year of pregnancy have an increased risk of poor perinatal outcomes with adverse neonatal health outcomes [60,61]. 

Pharmacotherapy may achieve weight loss that is intermediate between lifestyle modification and severe caloric restriction and/or surgical intervention. It has the potential to assist pre-pregnancy weight loss whilst maintaining nutritional requirements and is potentially a valuable adjunct to lifestyle modification for women with obesity attempting pre-pregnancy weight loss. As some medications have contraindications during pregnancy, this review will assess pharmacotherapy for weight loss prior to conception.

### 4.2. Pharmacotherapy for Pre-Pregnancy Weight Management

While there are several weight loss drugs currently available, there is little research on the effects of such drugs in the context of pre-pregnancy weight management, or the outcomes of subsequent pregnancies. It is essential to explore the effects of these drugs on maternal health and weight stability, as well as to assess suitable wash-out periods to avoid teratogenic effects on the developing fetus. Furthermore, whether pre-pregnancy weight loss with pharmacotherapy reduces the risk for the developmental programming of metabolic dysfunction and MAFLD is largely unknown.

Glucagon-like peptide-1 (GLP-1) receptor agonists are a class of drugs with great effects on appetite and metabolism. By potentiating the effects of endogenous GLP-1, a key incretin hormone, they limit postprandial glucose levels by potentiating the glucose-dependent insulin secretion from pancreatic β-cells [62]. By acting centrally GLP-1 also reduces hunger and promotes satiety [63]. Given that GLP-1RAs stimulate insulin production from the pancreas, influencing the metabolic function of the liver whilst promoting weight loss, GLP-1 receptor agonists are of interest as a pharmacotherapeutic strategy to improve maternal liver health and induce meaningful pre-pregnancy weight loss [64]. The LEAN clinical trial demonstrated the efficacy of the GLP-1RA liraglutide in MAFLD treatment; specifically, liraglutide induced the resolution of MASH in 39% of patients compared with 9% in the placebo group [65]. In addition, when liraglutide was given to mice prior to pregnancy, it induced weight loss and improved metabolic function in mothers, whilst improving their fertility rates [49]. Its preconception usefulness of preventing adverse fetal programming of metabolic health, including liver health in the offspring, has not yet been reported. 

Following the clinical success of GLP-1RAs as weight loss agents, synergistic drug combinations have been trialed to further improve their efficacy. The glucose-dependent insulinotropic polypeptide (GIP), which is another incretin hormone, regulates energy balance through cell-surface receptor signaling in the brain and adipose tissue. There is promising clinical data from co-agonists that allow for the concurrent activation of the GLP-1 and GIP known as GLP-1/GIP co-agonists. Recently, the GLP-1/GIP co-agonist, tirzepatide, was approved by the US Food and Drug Administration (FDA) for use in patients with obesity after it was found to induce substantial weight loss in the SURMOUNT-1 trial, inducing a 19.5% reduction in weight loss compared to a 3.1% reduction in patients receiving a placebo [66]. GLP-1 receptor agonists and GLP-1/GIP co-agonists are promising weight loss agents that could be valuable for pre-conception weight reduction to improve fertility and pregnancy outcomes and extend these benefits to the health of their offspring.

## 5. Conclusions and Future Directions

Both clinical and preclinical models of maternal obesity show that an obesogenic maternal environment fosters the progression of hepatic steatosis in the offspring, with an increased risk for MAFLD in adulthood. It is important that new interventional strategies are devised in the face of the obesity pandemic to improve intergenerational health outcomes. As the global rate of MAFLD rises, reducing levels of maternal obesity may be an effective strategy to lessen the burden of this disease. Pre-clinical models of maternal obesity have shown the promising potential of early pre-pregnancy weight modulation to reduce obesity rates and resultant MAFLD risk in adult offspring, but the current weight loss methods that are available for prospective mothers fall short. GLP-1RAs and GLP-1/GIP/glucagon have well-established weight loss benefits and could be considered for use in the context of maternal obesity, so long as there is sufficient time to induce weight loss, followed by a wash-out period prior to conception to avoid potential teratogenic effects if continued during gestation. The physiological effects of these drugs in a clinical setting of maternal obesity are inherently complex and thus, it is essential to discern their benefits in the context of pre-pregnancy weight modulation to reduce the intergenerational effects of maternal obesity on MAFLD in the offspring. 

## Figures and Tables

**Table 1:** Preclinical models of pre-pregnancy lifestyle modifications to improve liver health outcomes in offspring exposed to maternal obesity.

Ref	Authors	Year	Animal Species	Animal Model	Key Study Findings
[55]	Wesolowski et al.	2018	Japanese macaque	Maternal diet: chow (CON) or western-style (OB-WSD). A subset of OB-WSD mothers were switched to CON (OB-DR) approx. 9 weeks prior to and throughout pregnancy. Offspring age: Late third trimester fetuses	The offspring of OB-WSD mothers had increased hepatic steatosis, oxidative stress and expression of key regulators of de novo lipogenesis compared to the fetuses of CON mothers. Lipogenic gene expression and oxidative stress was reduced in fetuses of OB-DR mothers, although pre-pregnancy dietary switch did not induce complete normalization in the liver of fetuses.
[52]	Zhou et al.	2020	Mice (mixed background)	Maternal diet: normal-fat (NF) or high-fat diet (HFD). A subset of HFD mothers were switched to NF diet 9 weeks (H9N) or 1 week (H1N) prior to pregnancy. Offspring age and diet: Postnatal week 12, weaned to HFD.	The offspring of HFD and H1N mothers had increased hepatic steatosis, insulin resistance and expression of key regulators of de novo lipogenesis than the offspring of NF mothers. The offspring of H9N mothers had similar phenotypes to the offspring of NF mothers.
[53]	Hinrichs et al.	2023	C57Bl/6J mice	Maternal diet: CON or high-fat, fructose, cholesterol diet (HFFC). A subset had an exercise wheel introduced 2 weeks prior to pregnancy to allow for voluntary exercise (HFFC-Run). Offspring age and diet: Postnatal week 7, weaned to a HFFC diet.	The offspring of HFFC mothers had increased hepatic steatosis and fibrosis compared to the offspring of CON mothers. Hepatic fibrosis was reduced in the offspring of HFFC-Run mothers. Pre-pregnancy maternal exercise had no effect on hepatic steatosis in the offspring.
[54]	Kasper et al.	2021	C57BL/6N mice	Maternal diet: CON or WSD. A subset of WSD mothers had an exercise wheel introduced 2 weeks prior to pregnancy to allow for voluntary exercise (WSD-Run). Offspring age and diet: Postnatal week 3 (at weaning), or postnatal week 14, weaned to CON, but switched to WSD at postnatal week 5.	The offspring of WSD mothers had increased hepatic steatosis compared to the offspring of CON mothers. At postnatal week 3, the offspring of WSD-Run mothers had increased expression of regulators involved in hepatic energy metabolism compared to the offspring of WSD mothers. At postnatal week 14, the offspring of WSD-Run mothers had increased expression of key regulators of ß-oxidation, and inhibition of key regulators of lipogenesis in the liver compared to the offspring of WSD mothers.

## References

[1] World Health Organization (2021). World Health Organization Obesity and Overweight—Key Facts.

[2] Ogden C.L., Carroll M.D., Kit B.K., Flegal K.M. (2014). Prevalence of Childhood and Adult Obesity in the United States, 2011–2012. JAMA.

[3] Ehrenthal D.B., Maiden K., Rao A., West D.W., Gidding S.S., Bartoshesky L., Carterette B., Ross J., Strobino D. (2013). Independent Relation of Maternal Prenatal Factors to Early Childhood Obesity in the Offspring. Obstet. Gynecol..

[4] Dias M.d.S., Matijasevich A., Barros A.J.D., Menezes A.M.B., Schneider B.C., Hartwig F.P., Barros F.C., Wehrmeister F.C., Gonçalves H., Santos I.S. (2021). Influence of maternal pre-pregnancy nutritional status on offspring anthropometric measurements and body composition in three Brazilian Birth Cohorts. Public Health Nutr..

[5] Kopelman P.G. (2000). Obesity as a medical problem. Nature.

[6] Chalasani N., Younossi Z., Lavine J.E., Charlton M., Cusi K., Rinella M., Harrison S.A., Brunt E.M., Sanyal A.J. (2018). The diagnosis and management of nonalcoholic fatty liver disease: Practice guidance from the American Association for the Study of Liver Diseases. Hepatology.

[7] Younossi Z.M., Koenig A.B., Abdelatif D., Fazel Y., Henry L., Wymer M. (2016). Global epidemiology of nonalcoholic fatty liver disease—Meta-analytic assessment of prevalence, incidence, and outcomes. Hepatology.

[8] Blachier M., Leleu H., Peck-Radosavljevic M., Valla D.C., Roudot-Thoraval F. (2013). The burden of liver disease in Europe: A review of available epidemiological data. J. Hepatol..

[9] Hoyumpa A.M., Greene H.L., Dunn G.D., Schenker S. (1975). Fatty liver: Biochemical and clinical considerations. Am. J. Dig. Dis..

[10] Sanyal A.J. (2011). NASH: A global health problem. Hepatol. Res..

[11] Hagström H., Simon T.G., Roelstraete B., Stephansson O., Söderling J., Ludvigsson J.F. (2021). Maternal obesity increases the risk and severity of NAFLD in offspring. J. Hepatol..

[12] Bruce K.D., Cagampang F.R., Argenton M., Zhang J., Ethirajan P.L., Burdge G.C., Bateman A.C., Clough G.F., Poston L., Hanson M.A. (2009). Maternal high-fat feeding primes steatohepatitis in adult mice offspring, involving mitochondrial dysfunction and altered lipogenesis gene expression. Hepatology.

[13] McCurdy C.E., Bishop J.M., Williams S.M., Grayson B.E., Smith M.S., Friedman J.E., Grove K.L. (2009). Maternal high-fat diet triggers lipotoxicity in the fetal livers of nonhuman primates. J. Clin. Investig..

[14] Oben J.A., Mouralidarane A., Samuelsson A.-M., Matthews P.J., Morgan M.L., McKee C., Soeda J., Fernandez-Twinn D.S., Martin-Gronert M.S., Ozanne S.E. (2010). Maternal obesity during pregnancy and lactation programs the development of offspring non-alcoholic fatty liver disease in mice. J. Hepatol..

[15] Ashino N.G., Saito K.N., Souza F.D., Nakutz F.S., Roman E.A., Velloso L.A., Torsoni A.S., Torsoni M.A. (2012). Maternal high-fat feeding through pregnancy and lactation predisposes mouse offspring to molecular insulin resistance and fatty liver. J. Nutr. Biochem..

[16] Hammarstedt A., Gogg S., Hedjazifar S., Nerstedt A., Smith U. (2018). Impaired Adipogenesis and Dysfunctional Adipose Tissue in Human Hypertrophic Obesity. Physiol. Rev..

[17] Bugianesi E., Gastaldelli A., Vanni E., Gambino R., Cassader M., Baldi S., Ponti V., Pagano G., Ferrannini E., Rizzetto M. (2005). Insulin resistance in non-diabetic patients with non-alcoholic fatty liver disease: Sites and mechanisms. Diabetologia.

[18] Hellerstein M.K. (1999). De novo lipogenesis in humans: Metabolic and regulatory aspects. Eur. J. Clin. Nutr..

[19] Fabbrini E., Sullivan S., Klein S. (2010). Obesity and nonalcoholic fatty liver disease: Biochemical, metabolic, and clinical implications. Hepatology.

[20] Tsochatzis E., Papatheodoridis G.V., Archimandritis A.J. (2006). The Evolving Role of Leptin and Adiponectin in Chronic Liver Diseases. Off. J. Am. Coll. Gastroenterol..

[21] Zimmermann H., Trautwein C., Tacke F. (2012). Functional Role of Monocytes and Macrophages for the Inflammatory Response in Acute Liver Injury. Front. Physiol..

[22] Fernández-Sánchez A., Madrigal-Santillán E., Bautista M., Esquivel-Soto J., Morales-González Á., Esquivel-Chirino C., Durante-Montiel I., Sánchez-Rivera G., Valadez-Vega C., Morales-González J.A. (2011). Inflammation, Oxidative Stress, and Obesity. Int. J. Mol. Sci..

[23] Yan Y., Li S., Liu Y., Bazzano L., He J., Mi J., Chen W. (2019). Temporal relationship between inflammation and insulin resistance and their joint effect on hyperglycemia: The Bogalusa Heart Study. Cardiovasc. Diabetol..

[24] Kazankov K., Jørgensen S.M.D., Thomsen K.L., Møller H.J., Vilstrup H., George J., Schuppan D., Grønbæk H. (2019). The role of macrophages in nonalcoholic fatty liver disease and nonalcoholic steatohepatitis. Nat. Rev. Gastroenterol. Hepatol..

[25] Bessone F., Razori M.V., Roma M.G. (2019). Molecular pathways of nonalcoholic fatty liver disease development and progression. Cell. Mol. Life Sci..

[26] Heyens L.J.M., Busschots D., Koek G.H., Robaeys G., Francque S. (2021). Liver Fibrosis in Non-alcoholic Fatty Liver Disease: From Liver Biopsy to Non-invasive Biomarkers in Diagnosis and Treatment. Front. Med..

[27] Heerwagen M.J.R., Miller M.R., Barbour L.A., Friedman J.E. (2010). Maternal obesity and fetal metabolic programming: A fertile epigenetic soil. Am. J. Physiol.-Regul. Integr. Comp. Physiol..

[28] Barker D.J. (1990). The fetal and infant origins of adult disease. BMJ.

[29] Chang E., Hafner H., Varghese M., Griffin C., Clemente J., Islam M., Carlson Z., Zhu A., Hak L., Abrishami S. (2019). Programming effects of maternal and gestational obesity on offspring metabolism and metabolic inflammation. Sci. Rep..

[30] Ehrenberg H.M., Mercer B.M., Catalano P.M. (2004). The influence of obesity and diabetes on the prevalence of macrosomia. Am. J. Obstet. Gynecol..

[31] Moeckli B., Delaune V., Prados J., Tihy M., Peloso A., Oldani G., Delmi T., Slits F., Gex Q., Rubbia-Brandt L. (2022). Impact of Maternal Obesity on Liver Disease in the Offspring: A Comprehensive Transcriptomic Analysis and Confirmation of Results in a Murine Model. Biomedicines.

[32] Oddy W.H., Herbison C.E., Jacoby P., Ambrosini G.L., O’Sullivan T.A., Ayonrinde O.T., Olynyk J.K., Black L.J., Beilin L.J., Mori T.A. (2013). The Western Dietary Pattern Is Prospectively Associated With Nonalcoholic Fatty Liver Disease in Adolescence. Off. J. Am. Coll. Gastroenterol..

[33] Bellatorre A., Scherzinger A., Stamm E., Martinez M., Ringham B., Dabelea D. (2018). Fetal Overnutrition and Adolescent Hepatic Fat Fraction: The Exploring Perinatal Outcomes in Children Study. J Pediatr.

[34] Patel S., Lawlor D.A., Callaway M., Macdonald-Wallis C., Sattar N., Fraser A. (2016). Association of maternal diabetes/glycosuria and pre-pregnancy body mass index with offspring indicators of non-alcoholic fatty liver disease. BMC Pediatr..

[35] Ferré P., Foufelle F. (2010). Hepatic steatosis: A role for de novo lipogenesis and the transcription factor SREBP-1c. Diabetes Obes. Metab..

[36] Thompson M.D., Cismowski M.J., Trask A.J., Lallier S.W., Graf A.E., Rogers L.K., Lucchesi P.A., Brigstock D.R. (2016). Enhanced Steatosis and Fibrosis in Liver of Adult Offspring Exposed to Maternal High-Fat Diet. Gene Expr..

[37] Sanchez L.H.G., Tomita K., Guo Q., Furuta K., Alhuwaish H., Hirsova P., Baheti S., Alver B., Hlady R., Robertson K.D. (2018). Perinatal Nutritional Reprogramming of the Epigenome Promotes Subsequent Development of Nonalcoholic Steatohepatitis. Hepatol. Commun..

[38] Bernstein B.E., Meissner A., Lander E.S. (2007). The Mammalian Epigenome. Cell.

[39] Benhamouche-Trouillet S., Postic C. (2016). Emerging role of miR-21 in non-alcoholic fatty liver disease. Gut.

[40] Pirola C.J., Fernández Gianotti T., Castaño G.O., Mallardi P., San Martino J., Mora Gonzalez Lopez Ledesma M., Flichman D., Mirshahi F., Sanyal A.J., Sookoian S. (2015). Circulating microRNA signature in non-alcoholic fatty liver disease: From serum non-coding RNAs to liver histology and disease pathogenesis. Gut.

[41] Sugino K.Y., Mandala A., Janssen R.C., Gurung S., Trammell M., Day M.W., Brush R.S., Papin J.F., Dyer D.W., Agbaga M.P. (2022). Western diet-induced shifts in the maternal microbiome are associated with altered microRNA expression in baboon placenta and fetal liver. Front. Clin. Diabetes Healthc..

[42] Simino L.A., Panzarin C., Fontana M.F., de Fante T., Geraldo M.V., Ignácio-Souza L.M., Milanski M., Torsoni M.A., Ross M.G., Desai M. (2021). MicroRNA Let-7 targets AMPK and impairs hepatic lipid metabolism in offspring of maternal obese pregnancies. Sci. Rep..

[43] Zhang X., Liu S., Zhang C., Zhang S., Yue Y., Zhang Y., Chen L., Yao Z., Niu W. (2020). The role of AMPKα2 in the HFD-induced nonalcoholic steatohepatitis. Biochim. Biophys. Acta (BBA)-Mol. Basis Dis..

[44] Ceccarelli S., Panera N., Gnani D., Nobili V. (2013). Dual role of microRNAs in NAFLD. Int. J. Mol. Sci..

[45] Seki Y., Suzuki M., Guo X., Glenn A.S., Vuguin P.M., Fiallo A., Du Q., Ko Y.A., Yu Y., Susztak K. (2017). In Utero Exposure to a High-Fat Diet Programs Hepatic Hypermethylation and Gene Dysregulation and Development of Metabolic Syndrome in Male Mice. Endocrinology.

[46] Zhang W., Zhangyuan G., Wang F., Jin K., Shen H., Zhang L., Yuan X., Wang J., Zhang H., Yu W. (2021). The zinc finger protein Miz1 suppresses liver tumorigenesis by restricting hepatocyte-driven macrophage activation and inflammation. Immunity.

[47] Dodd J.M., Turnbull D.A., McPhee A.J., Wittert G., Crowther C.A., Robinson J.S. (2011). Limiting weight gain in overweight and obese women during pregnancy to improve health outcomes: The LIMIT randomised controlled trial. BMC Pregnancy Childbirth.

[48] Poston L., Bell R., Croker H., Flynn A.C., Godfrey K.M., Goff L., Hayes L., Khazaezadeh N., Nelson S.M., Oteng-Ntim E. (2015). Effect of a behavioural intervention in obese pregnant women (the UPBEAT study): A multicentre, randomised controlled trial. Lancet Diabetes Endocrinol..

[49] Rodrigo N., Chen H., Pollock C.A., Glastras S.J. (2022). Preconception weight loss improves fertility and maternal outcomes in obese mice. J. Endocrinol..

[50] Vega C.C., Reyes-Castro L.A., Bautista C.J., Larrea F., Nathanielsz P.W., Zambrano E. (2015). Exercise in obese female rats has beneficial effects on maternal and male and female offspring metabolism. Int. J. Obes..

[51] Carter L.G., Qi N.R., De Cabo R., Pearson K.J. (2013). Maternal exercise improves insulin sensitivity in mature rat offspring. Med. Sci. Sports Exerc..

[52] Zhou Y., Peng H., Xu H., Li J., Golovko M., Cheng H., Lynch E.C., Liu L., McCauley N., Kennedy L. (2020). Maternal diet intervention before pregnancy primes offspring lipid metabolism in liver. Lab. Investig..

[53] Hinrichs H., Faerber A., Young M., Ballentine S.J., Thompson M.D. (2023). Maternal Exercise Protects Male Offspring From Maternal Diet–Programmed Nonalcoholic Fatty Liver Disease Progression. Endocrinology.

[54] Kasper P., Breuer S., Hoffmann T., Vohlen C., Janoschek R., Schmitz L., Appel S., Fink G., Hünseler C., Quaas A. (2021). Maternal Exercise Mediates Hepatic Metabolic Programming via Activation of AMPK-PGC1α Axis in the Offspring of Obese Mothers. Cells.

[55] Wesolowski S.R., Mulligan C.M., Janssen R.C., Baker P.R., Bergman B.C., D’Alessandro A., Nemkov T., Maclean K.N., Jiang H., Dean T.A. (2018). Switching obese mothers to a healthy diet improves fetal hypoxemia, hepatic metabolites, and lipotoxicity in non-human primates. Mol. Metab..

[56] Furber C.M., McGowan L., Bower P., Kontopantelis E., Quenby S., Lavender T. (2011). Antenatal interventions for reducing weight in obese women for improving pregnancy outcome. Cochrane Database Syst. Rev..

[57] Sacha C.R., Page C.M., Goldman R.H., Ginsburg E.S., Zera C.A. (2018). Are women with obesity and infertility willing to attempt weight loss prior to fertility treatment?. Obes. Res. Clin. Pract..

[58] Purcell K., Sumithran P., Prendergast L.A., Bouniu C.J., Delbridge E., Proietto J. (2014). The effect of rate of weight loss on long-term weight management: A randomised controlled trial. Lancet Diabetes Endocrinol..

[59] Price S.A., Sumithran P., Nankervis A., Permezel M., Proietto J. (2019). Preconception management of women with obesity: A systematic review. Obes. Rev..

[60] de Rooij S.R., Painter R.C., Phillips D.I.W., Osmond C., Michels R.P.J., Godsland I.F., Bossuyt P.M.M., Bleker O.P., Roseboom T.J. (2006). Impaired Insulin Secretion After Prenatal Exposure to the Dutch Famine. Diabetes Care.

[61] Wax J.R., Cartin A., Wolff R., Lepich S., Pinette M.G., Blackstone J. (2008). Pregnancy Following Gastric Bypass Surgery for Morbid Obesity: Maternal and Neonatal Outcomes. Obes. Surg..

[62] Holst J.J. (2007). The Physiology of Glucagon-like Peptide 1. Physiol. Rev..

[63] Donnelly D. (2012). The structure and function of the glucagon-like peptide-1 receptor and its ligands. Br. J. Pharmacol..

[64] Svegliati-Baroni G., Patrício B., Lioci G., Macedo M.P., Gastaldelli A. (2020). Gut-Pancreas-Liver Axis as a Target for Treatment of NAFLD/NASH. Int. J. Mol. Sci..

[65] Armstrong M.J., Gaunt P., Aithal G.P., Barton D., Hull D., Parker R., Hazlehurst J.M., Guo K., Abouda G., Aldersley M.A. (2016). Liraglutide safety and efficacy in patients with non-alcoholic steatohepatitis (LEAN): A multicentre, double-blind, randomised, placebo-controlled phase 2 study. Lancet.

[66] Jastreboff A.M., Aronne L.J., Ahmad N.N., Wharton S., Connery L., Alves B., Kiyosue A., Zhang S., Liu B., Bunck M.C. (2022). Tirzepatide Once Weekly for the Treatment of Obesity. N. Engl. J. Med..

